# Genomic characterization of a unique Panton–Valentine leucocidin-positive community-associated methicillin-resistant *Staphylococcus aureus* lineage increasingly impacting on Australian indigenous communities

**DOI:** 10.1099/mgen.0.001172

**Published:** 2023-12-20

**Authors:** Joshua P. Ramsay, Nipuna Parahitiyawa, Shakeel Mowlaboccus, Christopher A. Mullally, Nicholas W.T. Yee, Princy Shoby, Elena Colombi, Hui-Leen Tan, Julie C. Pearson, Geoffrey W. Coombs

**Affiliations:** ^1^​ Curtin Medical School, Faculty of Health Sciences, Curtin University, Perth, WA, Australia; ^2^​ Curtin Health Innovation Research Institute, Faculty of Health Sciences, Curtin University, Perth, WA, Australia; ^3^​ Microbiology Department, Fiona Stanley Hospital, PathWest Laboratory Medicine, Murdoch, WA, Australia; ^4^​ Antimicrobial Resistance and Infectious Disease (AMRID) Research Laboratory, College of Science, Health, Engineering and Education, Murdoch University, Perth, WA, Australia

**Keywords:** conjugative mobilization, dfrG SXT, Panton–Valentine leucocidin, PVL-positive, ST5 CA-MRSA, Tn4791 Tn916 Tn7702 Tn553

## Abstract

In 2010 a single isolate of a trimethoprim-resistant multilocus sequence type 5, Panton–Valentine leucocidin-positive, community-associated methicillin-resistant *Staphylococcus aureus* (PVL-positive ST5 CA-MRSA), colloquially named WA121, was identified in northern Western Australia (WA). WA121 now accounts for ~14 % of all WA MRSA infections. To gain an understanding of the genetic composition and phylogenomic structure of WA121 isolates we sequenced the genomes of 155 WA121 isolates collected 2010–2021 and present a detailed genomic description. WA121 was revealed to be a single clonally expanding lineage clearly distinct from sequenced ST5 strains reported outside Australia. WA121 strains were typified by the presence of the distinct PVL phage φSa2wa-st5, the recently described methicillin resistance element SCC*mec*IVo carrying the trimethoprim resistance (*dfrG*) transposon Tn*4791*, the novel β-lactamase transposon Tn*7702* and the epidermal cell differentiation inhibitor (EDIN-A) plasmid p2010-15611-2. We present evidence that SCC*mec*IVo together with Tn*4791* has horizontally transferred to *Staphylococcus argenteus* and evidence of intragenomic movement of both Tn*4791* and Tn*7702*. We experimentally demonstrate that p2010-15611-2 is capable of horizontal transfer by conjugative mobilization from one of several WA121 isolates also harbouring a pWBG749-like conjugative plasmid. In summary, WA121 is a distinct and clonally expanding Australian PVL-positive CA-MRSA lineage that is increasingly responsible for infections in indigenous communities in northern and western Australia. WA121 harbours a unique complement of mobile genetic elements and is capable of transferring antimicrobial resistance and virulence determinants to other staphylococci.

## Data Summary

All Illumina sequence reads and genome assemblies completed with long-read data are available in National Center for Biotechnology Information (NCBI) BioProject PRJNA817299. A summary of genome features and metadata for sequenced isolates is presented in the Supplementary Material Tables S1, S2 and S3. DNA sequences of identified mobile genetic elements are provided in the Supplementary Material file Table S3, available in the online version of this article.

**Impact Statement**

Community-associated methicillin-resistant *Staphylococcus aureus* (CA-MRSA) is a significant cause of human disease worldwide. In 2010 a novel *S. aureus* multilocus sequence type 5 (ST5) lineage of CA-MRSA named WA121 was identified in Western Australia that carries the Panton–Valentine leucocidin (PVL) locus and uniquely the trimethoprim resistance gene *dfrG*. Strains with this combination of markers now represent ~14 % of MRSA cases in Western Australia and particularly impact on indigenous Australians. In this work we sequence 155 WA121 isolates spanning 2010–2021 and reveal that they form a monophyletic and clonally expanding lineage apparently unique to Australia. We provide a genomic description of WA121 and reveal that it harbours a unique repertoire of mobile genetic elements carrying virulence and antimicrobial resistance determinants, most of which are likely transferrable to other staphylococci through horizontal gene transfer. Importantly, this work confirms that the increasing numbers of reported *dfrG-*carrying, PVL-positive ST5-MRSA in northern Australia are likely a single clone. We also confirm that all WA121 isolates, despite being classified as sulfamethoxazole/trimethoprim (SXT) resistant by the Vitek 2 instrument, are SXT susceptible when tested by disc diffusion.

## Introduction

Over the last three decades, community-associated methicillin-resistant *Staphylococcus aureus* (CA-MRSA) has emerged globally. Although polyclonal, a small number of CA-MRSA clones are dominant in different regions of the world; for example, multilocus sequence type (ST) 8 carrying SCC*mec* type IV (ST8-IV, also known as USA300) in North America, ST80-IV in Europe and Northern Africa, ST59-IV/V in Asia, ST772-V and ST22-IV in the Indian subcontinent, ST93-IV in Australia/New Zealand and ST30-IV in the West Pacific region [1]. Transmission of the dominant clones in other regions has occurred, and characteristically they harbour the *lukS/F-PV* genes that encode the Panton–Valentine leucocidin (PVL) toxin [2].

In Australia, CA-MRSA infections first emerged in the Kimberley region of Western Australia (WA) in the early 1990s; primarily associated with Aboriginal people living in remote communities [3]. Since that time, the heavy burden of staphylococcal disease and an increasing prevalence of CA-MRSA in the Aboriginal populations across northern Australia have been reported [4]. First described in the early 2000s [5], ST93-IV, colloquially known as the ‘Queensland clone’ has become the predominant CA-MRSA clone across Australia [6], particularly in the indigenous population, where its dominance is believed to be linked to overcrowding [7], poor hygiene and inadequate healthcare [8]. Although known to cause severe infections, including necrotizing pneumonia, ST93-IV is typically associated with skin and soft tissue infections (SSTIs) [9].

In WA, colonization or infection with MRSA has been a notifiable condition since 1982, and since 1997 all MRSA isolates are referred to a central typing laboratory (PathWest Laboratory Medicine-WA) [10]. Over the last 10 years, a novel PVL-positive ST5-IV clone harbouring the trimethoprim resistance gene *dfrG* emerged as a frequent cause of SSTIs in remote aboriginal communities living in the northern regions of Australia [11, 12]. Colloquially known as WA121 MRSA, the clone was initially isolated in WA in 2010 from an abdominal abscess in a 62-year-old non-aboriginal male patient living in the Kimberley region [13]. Unlike other Western Australian clonal complex (CC) 5 clones, WA121 MRSA carries *edinA*, the epidermal cell differentiation inhibitor (EDIN-A) gene. From 1 July 2021 to 30 June 2022 1116 unique episodes of WA121 MRSA were identified in WA, accounting for 14 % of all MRSA referred to the central typing laboratory in that year. In comparison, the dominant CA-MRSA clone, PVL-positive ST93-IV, represented 27 % of all MRSA in the same year. The mean age of patients infected/colonized with WA121 MRSA in 2022 was 27 years (median 24 years). Although isolated in all Western Australian health regions, in 2021/2022 69 % of WA121 MRSA were isolated in the Kimberley health region (notification rate of 1127 per 100 000). The Kimberley region, located in the northern part of WA, encompasses an area of 424 517 km^2^, which is approximately three times the size of the United Kingdom. Approximately 50 % of the population identify as Aboriginal or Torres Strait Islander. In addition to multiple townships, there are over 100 Aboriginal communities of various population sizes scattered throughout the region.

It is unclear if WA121 MRSA represents a single genetic lineage and evolved from pre-existing methicillin-susceptible *S. aureus* present in WA or was introduced. Furthermore, the lack of molecular characterization of WA121 MRSA has exacerbated confusion surrounding its antimicrobial resistance. Automated laboratory susceptibility testing systems, including the Vitek 2, classify WA121 MRSA as sulfamethoxazole/trimethoprim (SXT) resistant. However, broth microdilution, disc diffusion and Etest results classify these trimethoprim-resistant *dfrG*-carrying ST5-IV isolates as SXT susceptible [11, 12]. The incorrect classification of WA121 as SXT resistant may have resulted in unwarranted restriction of oral SXT treatment for impetigo in remote Australian communities [11, 12, 14].

To gain an understanding of WA121 MRSA genetics and genomics and evaluate whether all designated WA121 PVL-positive ST5-IV isolates are of the same lineage, we performed whole-genome sequencing on 155 WA121 MRSA isolated over a 12 year period in the Kimberley region of WA. We confirmed that the isolates, together with PVL-positive ST5 strains isolated from the Northern Territory of Australia, form a monophyletic and clonally expanding lineage distinct from other reported (and sequenced) ST5 isolated worldwide. Long-read sequencing enabled us to create reference-quality assemblies for three of the WA121 isolates and we provide a detailed description of the typical genome features of WA121 that have likely played a role in its pathogenesis and clonal expansion.

## Methods

### Isolate collection

MRSA identified by the central typing laboratory at PathWest Laboratory Medicine-WA, Fiona Stanley Hospital as *mecA*/*nuc*/*lukF-PV* and *lukS-PV* PCR-positive, trimethoprim resistant and coagulase PCR/RFLP type 33 are designated as WA121 MRSA. The central typing laboratory performed multilocus sequence typing and SCC*mec* typing on the first WA121 MRSA isolated in WA (2010–15611), which was identified as ST5-IV [13]. From 1 July 2009 to 30 June 2021, 94 746 non-duplicate MRSA were referred to the central typing laboratory, of which 7589 (7.8 %) were characterized as WA121 MRSA. Overall, 1116 (14.7 %) WA121 MRSA were isolated in the Kimberley region, of which 57 % were from female patients. The median and mean age of patients living in the Kimberley with WA121 MRSA were 23 and 25 years, respectively. Five samples were isolated from blood culture and the remaining 1111 were isolated from skin and soft tissue infections (SSTIs). We selected a total of 155 WA121 MRSA isolated in the Kimberley region from 11 February 2010 to 15 July 2021 for analysis in this study. Except for the years 2010 and 2011, which had only 1 and 4 WA121 MRSA isolates, respectively, 15 WA121 MRSA isolated in each year were selected for sequencing. Generally, these were selected from the first part of the year, leading to some bias towards isolates obtained in the first quarter of each year. Overall, the 155 study isolates were distributed evenly over the 5 geographical locations within the Kimberley region and 57.4 % were isolated from female patients. Two selected samples were from blood infections and the remaining were from various SSTIs. The median (24 years) and the mean patient age (25 years) of the sequenced samples (Table S1) were also not significantly different from the total pool of WA121 MRSA isolated over the same period [15] (Table S2).

### Susceptibility testing

Antimicrobial susceptibility testing was performed on the Vitek 2 automated microbiology system using the AST-P612 panel and SXT susceptibility was confirmed by disc diffusion, according to the Clinical and Laboratory Standards Institute (CLSI) guidelines.

### Whole-genome sequencing

Short-read sequencing was performed on an Illumina NextSeq 500 platform (Illumina, USA) using 150 bp paired-end chemistry. Genomic DNA was extracted using the MagMAX automated platform as per the manufacturer’s instructions and 1 ng of DNA was used as input to the Nextera XT library preparation protocol. Long-read sequencing was performed using the MinION Mk1B system (ONT, UK) as previously described [16]. Long reads were base-called using Guppy (v6.0.1) super accuracy mode and assembled using Flye (v2.7.1). For strain 2010–15611 the minimum initial overlap in Flye was set to 10 000 bases to overcome assembly errors resulting from the second copy of Tn*7702* on plasmid p2010-15611-1. Assemblies were polished five times with long reads using Racon (GPU v1.4.15) and five times with Illumina reads using Pilon (v1.23). All Illumina sequence reads and genome assemblies completed with long-read data are available in National Center for Biotechnology Information (NCBI) BioProject PRJNA817299. The average and median depths of coverage for all Illumina reads were 212-fold and 217-fold, respectively (estimated by mapping reads to the 2010–15611 genome with bwa [17] and extracting statistics with Qualimap 2 [18]). Average depth of coverage for all Illumina reads are included in Table S1.

### Bioinformatic analyses

For genome analyses where assembled genomes were required the Illumina sequence reads were *de novo* assembled using SPAdes Genome Assembler v3.15.4 [19, 20] and assembled contigs were annotated using Prokka v1.14.6 [21]. Mobile genetic elements (MGEs) were identified manually using blastn searches for known elements and visualized using the Geneious software [22]. Closed genomes incorporating nanopore long reads were annotated with PGAP (doi: 10.1093/nar/gkaa1105). Resistance genes were detected using Abricate (Seemann T, Abricate, Github https://github.com/tseemann/abricate) and resfinder [23]. The presence of each identified MGE in each strain was determined by mapping unassembled Illumina sequence reads using srst2 with a custom gene database containing Tn*7702*, Tn*7491*, p2010-15611-1, p2010-15611-2, p2021-16354-1, φSa2wa-st5, φSaWA121-st5-1, φSaWA121-st5-2, SCC*mec*IVo and a pWBG751-like plasmid from strain 2017–22110 (sequences listed in Table S3). To detect multiple copies of MGEs in whole genomes Abricate was used with SPAdes-assembled genome contigs and an Abricate-compatible version of the MGE database described above.

### Phylogenetics

To construct whole-genome trees the Lyveset (v1.1.4f) [24] package was used to identify high-quality SNPs from short-read data using the completed ST5 WA121 strain 2010–15611 chromosome as a reference sequence. PHAST [25] was used to help identify potential phage regions in 2010–15611, which were then manually removed for use as a reference sequence. Lyveset was run using the --mask-phage and --mask-cliffs options. The Lyveset pipeline utilizes the following bioinformatics tools for construction of high-quality SNP trees from short-read sequence reads and complete genome sequences: CG-Pipeline [26], PHAST v1 [25], SMALT [27], Varscan 2 [28], bcftools [29] and RAxML v8 [30]. Completed genomes for 2010–15611, 2021–15363 and 2021–16354 were used instead of Illumina reads in construction of trees. *S. aureus* strains ED98, N315, SR130 and SR231 were used as outgroups. The resulting tree along with isolation location and phenotypic data were visualized using Microreact [31]. For construction of trees containing diverse CC5 isolates, the same phage-deleted 2010–15611 reference sequence was used and maximum-likelihood trees were constructed using Lyveset. beast 2 [32] (v2.7.5 with -beagle_GPU option [33]) was additionally used (10 000 trees compared) on the same identified SNP alignments to confirm the topology of maximum-likelihood trees using Bayesian analysis and 95 % of these trees shared the same topology as the maximum-likelihood tree.

### Conjugation experiments

Conjugation experiments were carried out using the BHIB-PEG broth mating method as previously described [34]. Exconjugants were selected for on tryptone soy agar (TSA) containing rifampicin (25 µg ml^−1^), fusidic acid (5 µg ml^−1^) and 80 µM cadmium. Donor numbers were estimated on medium containing cadmium only.

## Results and discussion

### Overview of the selected WA121 representatives

Isolates analysed in this work included the first and only WA121 isolate from 2010 (WA121 2010–15611) and all four WA121 isolates from 2011. For the years 2012 to 2021, 15 isolates were chosen from each year across 6 locations in the Kimberley region (Broome, Derby, Fitzroy Crossing, Halls Creek, Wyndham, Kununurra). Aside from two isolates sourced from blood, all isolates were cultured from SSTIs, often associated with abscess and pus formation (sites of infection listed in Table S2). All 155 isolates were classified as SXT resistant by the Vitek 2 automated system but all were found to be SXT susceptible by disc diffusion. Apart from three erythromycin-resistant isolates and a single isolate that was resistant to ciprofloxacin, all 155 WA121 isolates were sensitive to all other antimicrobials tested (Table S4).

### WA121 is a clonally expanding PVL-positive ST5-IVo strain unique to Australia

**Fig. 1.**
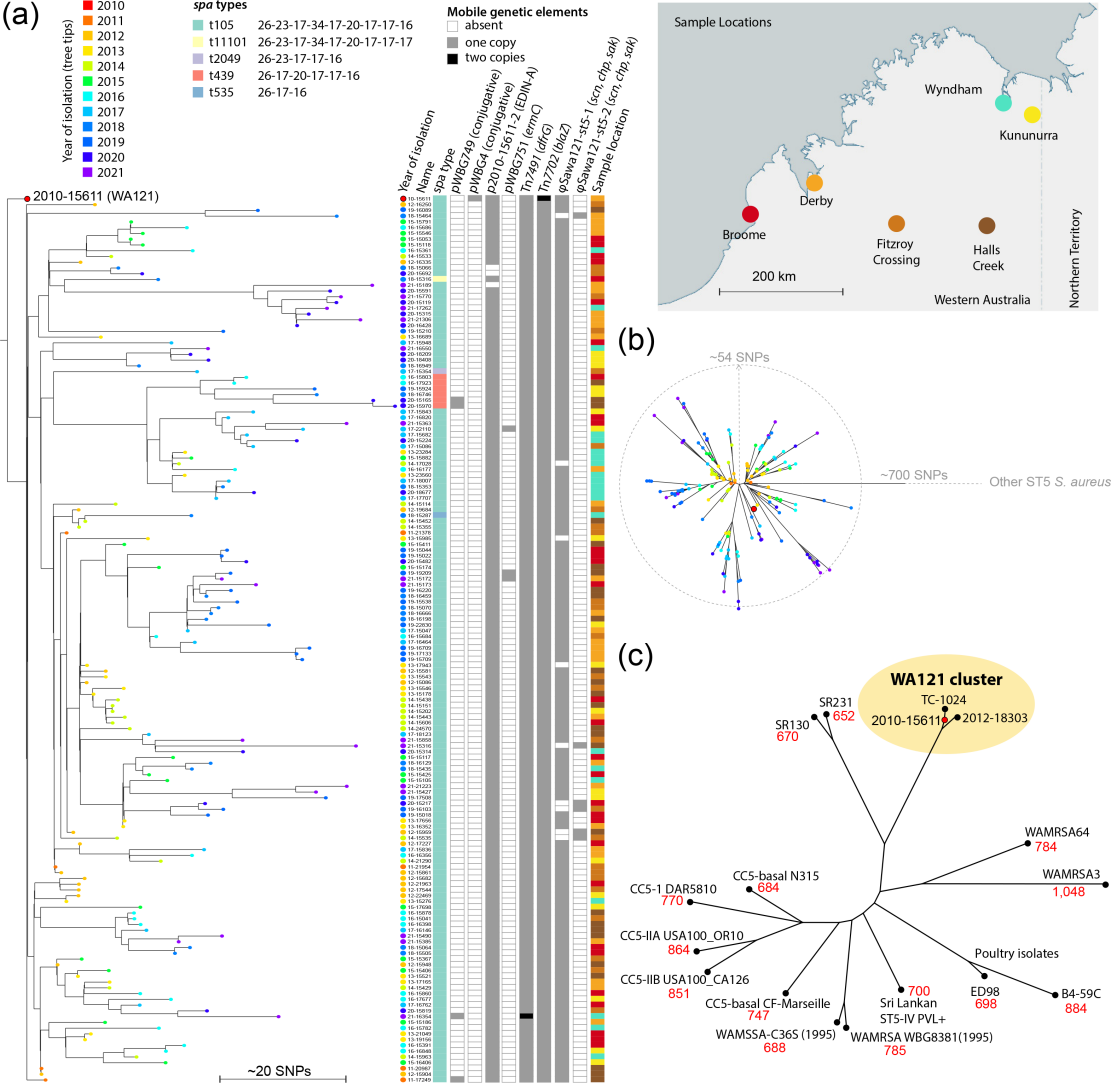
Phylogenetic analysis and genome features of WA121 isolates. (**a**) Comparison of all sequenced WA121 isolates. High-quality SNP calling was carried out with the Lyveset pipeline, using WA121 2010–15611 as the reference. (**a**) A rooted tree constructed from 155 WA121 isolates using *S. aureus* ST5 strains SR130, SR231, N315 and ED98 as outgroups (not shown). Strain 2012–18303 was positioned basal to 2010–15611 and was therefore removed for clarity. Branch tips are coloured according to year of isolation. To conserve space, isolate names have been abbreviated by removing the leading ‘20’ from each name, e.g. 2010–15611 is labelled as 10–15611. Distinctive metadata for each isolate are shown to the right of the tree, indicating geographical location of origin, *spa* type and presence of mobile elements, as indicated in the key above. All isolates carried the PVL phage φSa2wa-st5 [44] and SCC*mec*IVo [12] (Table S1, not shown in figure). (**b**) An unrooted version of tree in (a) to illustrate the clonal expansion of WA121 isolates with nodes coloured by year of isolation as in (a). (**c**) Unrooted tree constructed from high-quality SNPs identified between 2010–15611, 2012–18303 and diverse CC5/ST5 isolates as described in the text. TC-1024 (an abbreviation for TRIST CASE 1024) is a representative of a clonal outbreak of PVL-positive ST5-SCC*mec*IVo identified in the Australian Northern Territory and is part of the WA121 cluster described here. SNP differences between isolates and WA121 2010–15611 are shown in red text under each strain label.

The genome of the first WA121 strain isolated in WA (2010–15611) was further sequenced using nanopore long-read sequencing technology to enable construction of a high-quality reference sequence for annotation and phylogenetic analysis (Fig. 1). The assembled 2 840 066 bp chromosome of 2010–15611 was used as a reference sequence together with short-read sequences for other isolates to identify single-nucleotide polymorphisms (SNPs) and generate a maximum-likelihood tree (Fig. 1a). For this, Lyveset [24] was used, which is a pipeline for the conservative detection and scoring of high-quality SNPs using a reference genome and includes automated cleaning and trimming of reads, masking of phage genomes and masking of SNPs within areas of lower coverage. Overall, 153 of the other WA121 isolates differed by fewer than 59 high-quality SNPs from 2010 to 15611. The remaining isolate, 2012–18303 differed from all WA121 other isolates by at least 69 SNPs. *In silico* multilocus sequence typing confirmed that 154 of the WA121 isolates were designated ST5 (Table S1) and the remaining isolate 2015–15117 was a single-locus variant, ST7551 (Fig. 1a).

A clear pattern of clonal expansion was observed for the 2011–2021 period (Fig. 1b), with the first isolated WA121 (2010–15611) positioned at the base of the tree. WA121 isolated between 2011 and 2012 were on average 13 SNPs different from 2010 to 15611 (*n*=18, σ=3), whilst the 2021 isolates were on average 42 SNPs different from 2010 to 15611 (*n*=15, σ=12). Only isolate 2012–18303 was positioned more basally than 2010–15611, suggesting that 2012–18303 is related to an ancestor of 2010–15611 and may have diverged prior to the major clonal expansion observed for this set of isolates. The majority of WA121 isolates (144/155) harboured the *S. aureus *-specific staphylococcal protein A type (s*pa* type) t105. The remaining nine isolates carried *spa* types containing deletions relative to the t105 repeat sequence and are thus likely derived from t105. There was no obvious correlation between the clustering of isolates in the tree and the geographical location. For instance, isolates identified with *spa* type t439 appeared in both Broome and Halls Creek in 2016 (and in Kununurra in 2019), locations that are separated by a road distance of 685 km. In summary, the 155 WA121 isolates belong to a single clonally expanding lineage that has likely descended from a common ancestor related to the first WA121 isolated in 2010.

To place WA121 within the broader phylogenetic context with other more diverse *S. aureus* CC5 strains, a second SNP tree was constructed using representatives of CC5 subclades CC5-basal (N315 and CF-Marseille), CC5-I (DAR5810), CC5-IIA (USA100_OR-10) and CC5-IIB (USA100_CA126) [35]. We also included ST5 MRSA and methicillin-susceptible *S. aureus* (MSSA) strains previously isolated in WA (WAMRSA3, WAMRSA64, 1995-MRSA-WBG8381 [16] and 1995-MSSA-C36S [36]), a PVL-positive ST5-IVo isolate from the Northern Territory of Australia (TC-1024 [12]), a representative of an emerging Sri Lankan PVL-positive ST5 clade (strain 229895 [37]) and the poultry-associated ST5 isolates ED98 and B4-59C. ST5 isolates SR130 and SR231 [38] were also included since they were frequently identified as the top hits in blastn searches using regions of the 2010–15611 chromosome sequence as a query. The constructed maximum-likelihood tree indicated the WA121 lineage forms a distinct cluster together with the PVL-positive ST5-IVo Northern Territory isolates (represented by TC-1024) [12]. In this tree the WA121 cluster is separated from ST5 CA-MRSA and CA-MSSA previously isolated in WA and all other CC5/ST5 strains included in this comparison. There was an estimated minimum of 652 SNPs between the WA121 cluster and all other CC5 representatives. The NCBI Pathogen Detection Isolate database (beta) (https://www.ncbi.nlm.nih.gov/pathogens/), which on 10 August 2023 included 105 748 *S *. *aureus* isolates, also supports WA121 being a distinct clone (SNP cluster PDS000133861.7) containing 229 sequences (including those generated in this work). Collectively, the PDS000133861.7 cluster isolates differ from each other by 38 SNPs on average and a maximum of 94 SNPs. These isolates almost exclusively originate from WA, Australian Northern Territory, or other parts of Australia, and only a single isolate has been reported outside Australia (strain D2190, Germany [12, 39]).

### The unique WA121 mobilome

**Fig. 2.**
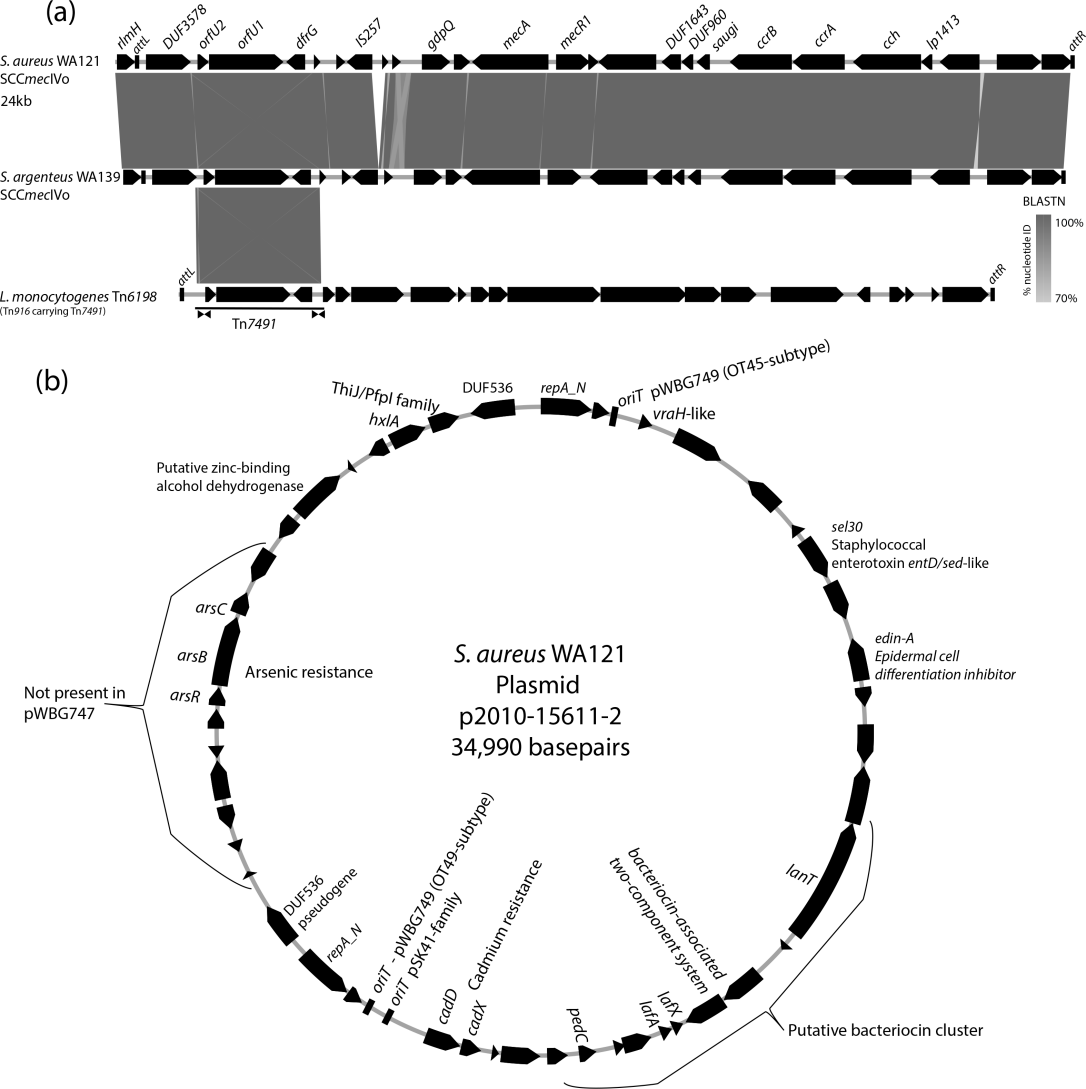
SCC*mec*IVo and p2010-15611-2. (**a**) Gene maps for SCC*mec*IVo are shown and a blastn comparison with a copy of SCC*mec*IVo identified in *S. argenteus* WA139 and the first-identified copy of the *dfrG* transposon Tn*4791* within the *L. monocytogenes* integrative and conjugative element Tn*6198*. (**b**) A gene map of plasmid p2010-15611-2 (from isolate 2010–15611) with putative gene names and/or functions indicated.

All 155 sequenced WA121 isolates harbour SCC*mec*IVo. SCC*mec*IVo is defined as a variant of SCC*mec*IVc containing a 3.3 kb *dfrG* resistance transposon (Fig. 2a). This transposon was originally identified in *Listeria monocytogenes* inserted within a variant (Tn*6198*) of the integrative and conjugative element Tn*916* [40]. For clarity of reference, we registered the *dfrG* transposon here with the designation Tn*7491* (https://transposon.lstmed.ac.uk). As described previously [40], Tn*7491* possesses the terminal inverted repeat sequence 5′- TAAGTAGTTCAGTTTTGGAGTACAAAA-3′ at each end and is flanked by a 9 bp direct repeat/target site duplication. We detected evidence of a second copy of Tn*7491* in the 2021–16354 genome sequence and so completed this genome using long-read nanopore sequencing. WA121 isolate 2021–16354 harboured a second copy of Tn*7491* between the convergently orientated *csbB* and *saeS* genes. The second Tn*7491* contained a deletion beginning within its 3′-end comprising 10 bp of Tn*7491* and 9 bp of chromosomal DNA, which removed any Tn*7491* target site duplication that may have existed at the Tn*7491* chromosome junction.

During our interrogation of trimethoprim-resistant staphylococcal isolates in the central typing laboratory collection (PathWest Laboratory Medicine-WA) we identified a *Staphylococcus argenteus* isolate (WA139) originating from a Kimberley region patient in 2014 that carried SCC*mec*IV and the *dfrG* gene, so we completed the genome sequence of this isolate using Oxford Nanopore and Illumina sequencing. Like the WA121 *S. aureus* isolates, *S. argenteus* WA139 carried a copy of SCC*mec*IVo together with its *dfrG* transposon Tn*7491* (Fig. 2). The Tn*7491* copy was present in the same position within the SCC*mec* as in WA121 genomes. The *S. argenteus* SCC*mec*IVo was identical to that in WA121 aside from a 355 bp deletion directly adjacent to the terminal inverted repeat sequence for IS*257* (5′-ACTTTGCAACAGAACC-3′). Overall, these observations indicate that the *dfrG* transposon Tn*7491* retains an ability to copy itself to new locations in the *S. aureus* genome and can also horizontally transfer together with SCC*mec*IVo between *Staphylococcus* spp.

All but three WA121 isolates (2018–15066, 2020–15692 and 2021–15189) carried a copy of a 34 990 bp plasmid named p2010-15611-2 (named after isolate 2010–15611) encoding the epidermal cell differentiation inhibitor (EDIN-A) gene *edinA* [41, 42]. p2010-15611-2 also carries the *entD*-like enterotoxin allele *sel30*, a lactacin F (*lafX/lafA*)-like bacteriocin cluster, cadmium resistance genes *cadDX*, and an arsenic resistance cluster (*arsRBC*). p2010-15611-2 carries three origin-of-transfer (*oriT*) sequences that mimic those used by conjugative plasmids pWBG749, pWBG745 and pSK41. p2010-15611-2 closely resembles the 33.7 kb plasmids pWBG747 and pWBG746 (accessions GQ900399 and GQ900390), which were isolated in ST75 MSSA and MRSA collected in the Kimberley area in 1995 and have been demonstrated to be horizontally mobilizable by conjugative plasmid pWBG749. pWBG747 and pWBG746, however, lack the arsenic resistance cluster and instead carry a 3748 bp region containing type III restriction modification genes. It seems possible that the pWBG747/pWBG746 and p2010-15611-2 have a direct common ancestor; however, similar plasmids (including those retaining the arsenic resistance gene cluster) are well distributed internationally. blastn hits for p2010-15611-2 identified similar plasmids in strains isolated from wastewater in South Africa (CP060279), human skin in Washington, USA (CP116910) and the ‘EDIN-A’ plasmid of *S. aureus* E-1 [43] (AP003089.1). The top blastn hit for p2010-15611-2 (99.94 % identity with 100 % coverage) was to a 47 kb plasmid present in the ATCC29213 Wichita strain (ST5 MSSA), which is used as a control strain in antimicrobial susceptibility testing.

The PVL locus in all WA121 isolates is present within the prophage φSa2wa-st5. φSa2wa-st5 is a recently described [44] family *Siphoviridae* bacteriophage of the genus *3alikevirus* [45]. φSa2wa-st5 is inserted at the 3′-end of a DUF1672 lipoprotein gene. The *scn*, *chp* and *sak* innate immune evasion cluster genes are encoded within the 3′-end of an identified 44 kb β-haemolysin-converting bacteriophage named here φSawa121-st5-1 (CP093935 : 2061311–2104944). blastn searches suggest that this prophage is most like φBU01 (98 % identity, 85 % coverage) [46]. φSawa121-st5-1 was found inserted into the *hlb* gene and flanked by imperfect directly repeated sequences AACGTTTATATGTTATCGA and AAGGTTTCTATGTATCCGA. φSawa121-st5 was detected in 146 of the WA121 genomes. Six of the remaining isolates were found to carry a distinct 43 kb β-haemolysin-converting bacteriophage named here φSawa121-st5-2 (Table S1 and Table S3). φSawa121-st5-2 is flanked by the same imperfect direct repeats as φSawa121-st5 but appears most like the PVL-phage φSa2-NARSA676 (OP493558 : 98 % identity over 72 %) but carries the *scn*, *chp* and *sak* genes within its 3′-end instead of PVL genes. The remaining three isolates lacked the *scn*, *chp* and *sak* but possessed an intact *hlb* β-haemolysin gene. All isolates harboured the enterotoxin gene cluster (*seg*, *sei*, *selm*, *seln*, *selo* and *selu*) and copies of the *selw* and *selx* enterotoxin-like genes at distinct chromosomal locations (Supplementary Material).

**Fig. 3.**
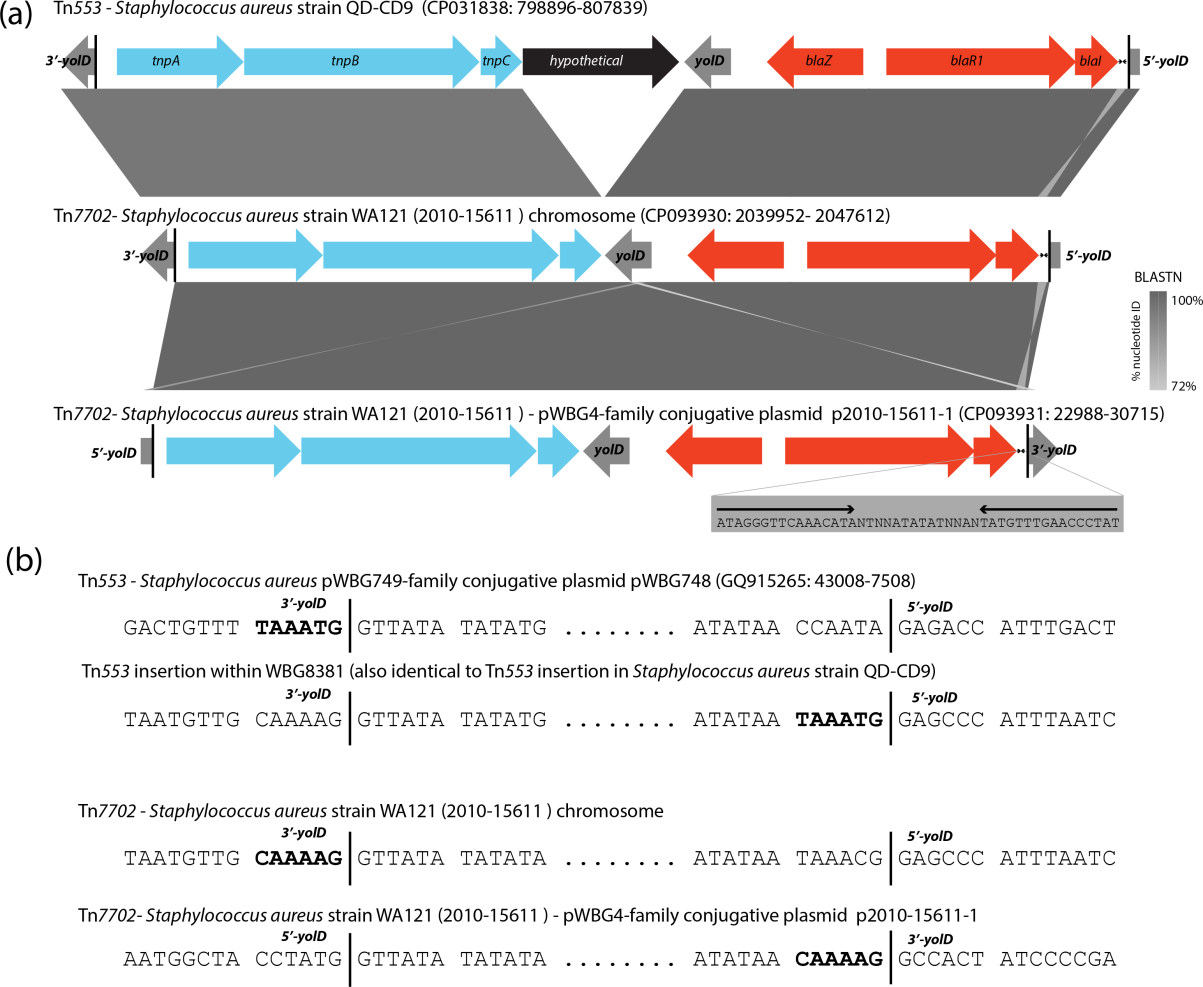
A novel variant of the Tn*553* β-lactamase transposon, Tn*7702*. (**a**) A blastn alignment of the Tn*553* and Tn*7702* transposons was created using Easyfig [56], with shading indicating sequence similarity between elements, as indicated in the key on the right. Vertical black bars indicate the transposon/chromosome junctions at the chromosomal copy of the *yolD* gene. An inverted repeat is present at the right end of Tn*553* and Tn*7702* between the *blaI* gene and the right-end junction (sequence indicated in the grey box). Coordinates and sequence accessions are listed above each element diagram. (**b**) DNA sequence junctions present at each end of copies of Tn*553* on pWBG748 and the WBG8381 chromosome and Tn*7702* in the WA121 chromosome and the p2010-15611-1 conjugative plasmid are shown, with black vertical lines indicating the Tn sequence borders. The bold sequences indicate the 6 bp sequences potentially copied from one insertion location to another during transposition [47].

All WA121 isolates were found to carry a β-lactamase transposon related to Tn*553*, registered in this work as Tn*7702* (https://transposon.lstmed.ac.uk) (Fig. 3). Tn*553* is a recently described transposon related to the *ermA* transposon Tn*554*, but instead of *ermA* Tn*554* carries the *blaZ-blaR1-blaI* region homologous with that carried by Tn*552* [47]. Tn*553* is found inserted within divergent *yolD* genes present in staphylococcal chromosomes and plasmids. The function of *yolD* is unknown, although it appears to have some genetic association with error-prone polymerases on various mobile genetic elements [48]. Tn*553* itself carries a distinct copy of the *yolD* gene, possibly complementing the *yolD* disruption caused by Tn*553* insertion. The Tn*7702* transposon found in all WA121 isolates is 7661 bp in length and shares 81 % nucleotide identity with Tn*553* over aligned regions (Fig. 3a). Compared to the 9050-bp Tn*553* sequence, Tn*7702* contains a deletion between the *tnpC* gene and the *yolD* gene, which removes a hypothetical open reading frame present only on Tn*553*.

Serial transposition experiments with Tn*553-*related transposons reveal that they exhibit a molecular phenomenon in which the last 6 bp of sequence at the 3′-end of the transposon is replaced by a new 6 bp sequence following each transposition event [47, 49–51]. This new 6 bp sequence is a copy of the 6 bp sequence present immediately upstream of the previous insertion site (Fig. 3b). We observed evidence for this phenomenon for two copies of Tn*553* in ST5 strain WBG8381. WBG8381 is the original identified host of the pWBG749 conjugative plasmid [16, 52] and harbours a copy of Tn*553* within the chromosomal copy of the *yolD* gene (CP071046 : 2027462–2018413). The original WBG8381 isolate carried two near-identical conjugative plasmids, pWBG748 and pWBG749 (Frances O’Brien personal communication). pWBG748 differs from pWBG749 in that it carries a copy of Tn*553* within the pWBG748-encoded *yolD* gene (GQ915265) [47]. Interestingly, the 6 bp sequence directly 5′ of the left Tn*553* junction on pWBG748 contains the 5′-TAAAG-3′ sequence and this same sequence is present directly 5′ of the right-hand Tn*553* junction in the WBG8381 chromosome, suggesting that the chromosomal Tn*553* copy may have been copied from pWBG748 (Fig. 3b).

WA121 2010–15611 was found to carry an additional pWBG4-like conjugative plasmid, p2010-15611-1, which harbours a conjugation gene cluster related to those present on various staphylococcal linezolid resistance plasmids [53]. Analysis of p2010-15611-1 revealed that it carried a second copy of the Tn*553-*like transposon Tn*7702* inserted within the p2010-15611-1 *yolD* gene. The Tn*7702* copy in p2010-15611-1 was inserted in the reverse orientation and at a distinct position within the *yolD* gene compared to documented Tn*553* insertion sites and is also distinct from the insertion site of the 2010–15611 chromosomal copy of Tn*7702* [47]. Further inspection revealed the right-hand junction of the p2010-15611-1 Tn*7702* copy carried the sequence CAAAAG, which is identical to the 6 bp immediately 5′ of the chromosomal Tn*7702* insertion site. Therefore, the p2010-15611-1 plasmid Tn*7702* copy may have been copied from the WA121 chromosome Tn*7702* copy. In summary, Tn*553* and the unique Tn*7702* variant described here may exploit two distinct families of conjugative plasmids for horizontal transfer through insertion in plasmid-borne *yolD* genes. pSK41 family plasmids also carry conserved *yolD* homologues (WP_011117677) [54], so it is tempting to speculate they may also help disseminate Tn*553* and Tn*7702* into new *S. aureus* hosts. It remains unclear how these transposons target the *yolD* gene for insertion, given the low sequence identity shared by various plasmid and chromosomal *yolD* genes [47] and the apparent lack of site/orientation specificity observed for the p2010-15611-1 copy of Tn*7702*.

In summary, the WA121 genomes analysed all carry the SCC*mec*IVo methicillin resistance element containing the *dfrG* transposon Tn*7491*. They all carry a chromosomal copy of a novel β-lactamase transposon Tn*7702* and all but three carry the epidermal cell differentiation inhibitor A (EDIN-A) plasmid p2010-15611-2. All WA121 isolates harbour the PVL locus on phage φSa2wa-st5 [44]. Aside from β-lactam and trimethoprim resistance, most isolates were susceptible to other antimicrobials, aside from three erythromycin-resistant isolates carrying a 2.2 kb *repL*/*ermC* resistance plasmid resembling pWBG751 (CP070988).

### A WA121 isolate carrying a pWBG749-like conjugative plasmid can mobilize the EDIN-A plasmid p2010-15611-2 by conjugation

The EDIN-A plasmid p2010-15611-2 carries *oriT* sequences resembling those of conjugative plasmids pWBG749, pWBG745 and pSK41 (Fig. 1a). These *oriT* are identical to those present on pWBG747, which is capable of being mobilized into diverse *S. aureus* lineages by the conjugative plasmid pWBG749. Amongst the 155 isolates sequenced here, we detected pWBG749-like conjugative plasmids in 4 isolates (isolates 2011–17249, 2020–15165, 2020–15970 and 2021–16354)

To test whether p2010-15611-2 was transferrable by conjugative mobilization, isolate 2021–16354, which carries a pWBG749 family conjugative plasmid (CP093936) and a copy of the EDIN-A p2010-15611-2 plasmid (CP093937), was used as a donor in conjugation experiments where cadmium resistance was used to select for transfer of p2010-15611-2. As a control, isolate 2021–15363, which only carries a copy of the p2010-15611-2 plasmid (CP093934), was also used as a donor. The fusidic acid and rifampicin-resistant *S. aureus* strain WBG541 was used as a recipient. Only isolate 2021–16354 carrying the pWBG749 family conjugative plasmid was able to mobilize p2010-15611-2 (Table 1). We also tested whether isolate 2010–15611, which carries the pWBG4 family conjugative plasmid, was able to mobilize p2010-15611-2, but did not observe any p2010-15611-2 transfer.

**Table 1.** Mobilization of plasmid p2010-15611-2

Donor strain	Conjugative plasmid	Conjugative plasmid family	p2010-15611-2 Mobilization frequency
2021–16354	p2021-16354-1	pWBG749-family	2.35×10^−6^*
2010–15611	p2010-15611-1	pWBG4-family	<1. 00×10^−8^†
2021–15363	None		<1. 00×10^−8^†

*Exconjugants per donor averaged from three biological replicates.

†Limit of detection.

### Conclusions

Several reports have documented increasing numbers of PVL-positive ST5-MRSA isolated from infections in individuals located in the northern and western parts of Australia [11–13, 15, 55]. However, it has been unclear if these isolates are of a single lineage and if they are related to other PVL-positive ST5-MRSA isolated elsewhere. In this work we clearly define the WA121 clade as a single clonal lineage unique to Australia. Following the initial emergence of WA121 in ~2010 the incidence of WA121 infections has steadily increased and is now responsible for a significant proportion of infections, particularly in the parts of Australia inhabited by the highest proportions of indigenous Australians.

WA121 harbours a distinct and largely invariant ensemble of mobile genetic elements, many of which have only recently been defined or exposed in this work. This includes the PVL phage φSa2wa-st5, two novel β-haemolysin-converting phage carrying the human innate immune evasion genes *scn*, *chp* and *sak*, the recently defined SCC*mec*IVo element carrying the *dfrG* resistance transposon Tn*7491* and a new β-lactamase transposon Tn*7702*. All but three WA121 carried the EDIN-A plasmid p2010-15611-2, which likely enhances the ability of WA121 to cause soft-tissue infections. We also presented evidence that several of these mobile elements have retained their mobility. SCC*mec*IVo, together with Tn*7491*, appears to have transferred horizontally to *S. argenteus *. Tn*7702*, like the related Tn*553* transposon, inserts into the *yolD* genes of conjugative plasmids, presumably facilitating its horizontal distribution. Finally, we demonstrated that natural WA121 isolates harbouring variants of the pWBG749 conjugative plasmid mobilize transfer of the EDIN-A plasmid p2010-15611-2. Therefore, WA121 is not only a direct burden on human health but is likely be capable of enhancing the virulence and resistance phenotypes of other staphylococci through horizontal gene transfer.

In summary, WA121 is a trimethoprim-resistant, ST5 PVL-positive MRSA that appears to be unique to Australia. It seems possible that this lineage arrived or evolved some time shortly before 2010. However, given the distinct deep-rooted phylogenetic position of WA121 within clonal complex 5 and the lack of closely related strains isolated (and sequenced) elsewhere, it is difficult to speculate with any certainty from where WA121 originated. Little is known about the colonization rates of the Australian population by WA121; however, given the rapid emergence of WA121 as a cause of SSTIs in northern and western Australian indigenous communities, it would seem that it is well adapted to these populations and environmental conditions. Increased community surveillance of colonized individuals and molecular interrogation of WA121 colonization and pathogenesis factors may help us understand why it has become so successful and how we might implement strategies to restrict the disease burden of WA121.

## Supplementary Data

Supplementary material 1
