# Corrigendum: Genomic characterisation of a unique Panton-Valentine leucocidin-positive community-associated methicillin-resistant Staphylococcus aureus lineage increasingly impacting Australian indigenous communities

**DOI:** 10.1099/mgen.0.001192

**Published:** 2024-01-31

**Authors:** Joshua P. Ramsay, Nipuna Parahitiyawa, Shakeel Mowlaboccus, Christopher A. Mullally, Nicholas W.T. Yee, Princy Shoby, Elena Colombi, Hui-Leen Tan, Julie C. Pearson, Geoffrey W. Coombs

**Affiliations:** ^1^​ Curtin Medical School, Faculty of Health Sciences, Curtin University, Perth, WA, Australia; ^2^​ Curtin Health Innovation Research Institute, Faculty of Health Sciences, Curtin University, Perth, WA, Australia; ^3^​ Microbiology Department, Fiona Stanley Hospital, PathWest Laboratory Medicine, Murdoch, WA, Australia; ^4^​ Antimicrobial Resistance and Infectious Disease (AMRID) Research Laboratory, College of Science, Health, Engineering and Education, Murdoch University, Perth, WA, Australia

## Full-Text

In the published version of this article an error was identified regarding Fig. 1b and in the third paragraph of the results section.

 

**Fig. 1.**
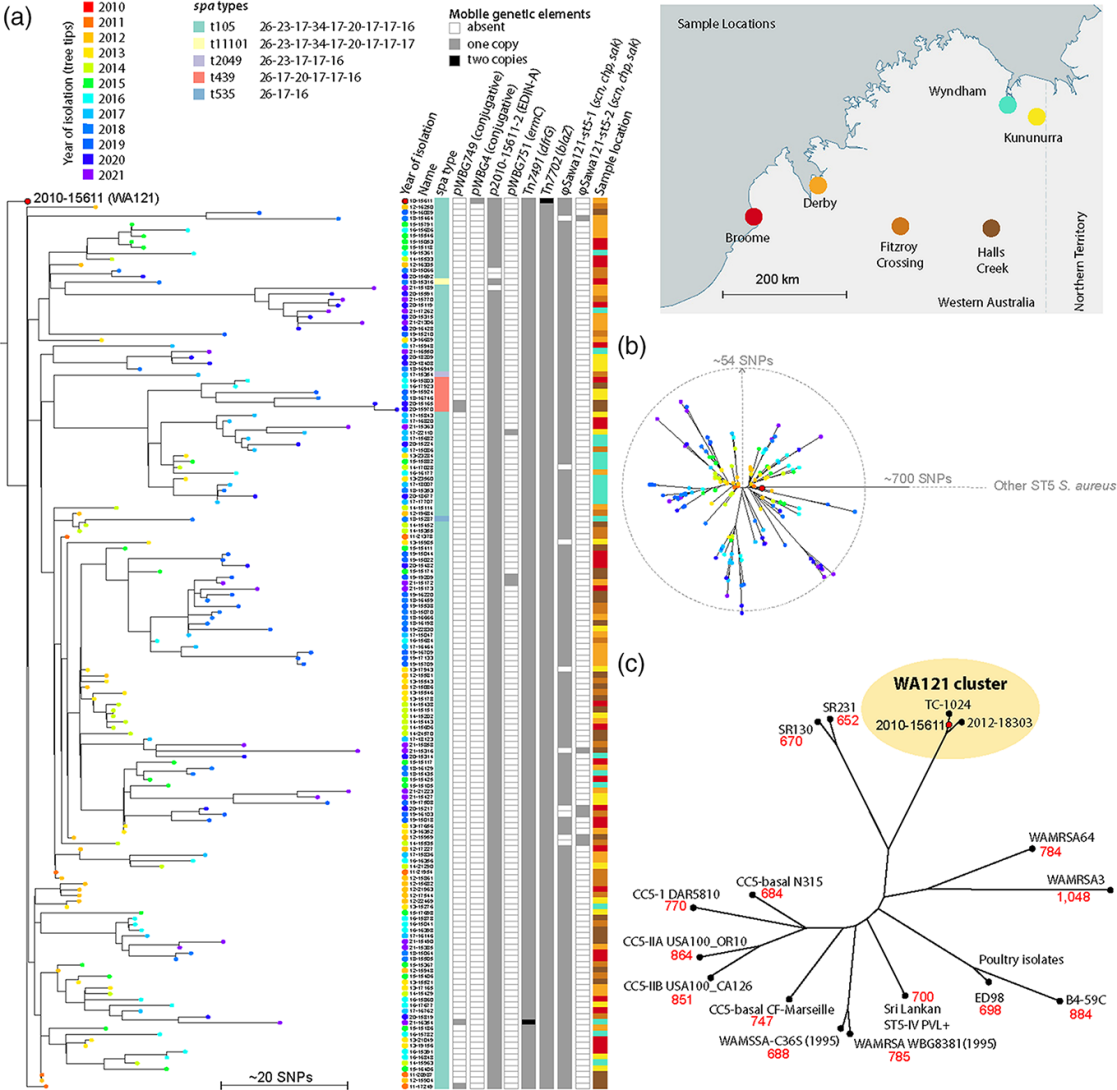
Phylogenetic analysis and genome features of WA121 isolates. (a) Comparison of all sequenced WA121 isolates. High-quality SNP calling was carried out with the Lyveset pipeline, using WA121 2010–15611 as the reference. (a) A rooted tree constructed from 155 WA121 isolates using *S. aureus* ST5 strains SR130, SR231, N315 and ED98 as outgroups (not shown). Strain 2012–18303 was positioned basal to 2010–15611 and was therefore removed for clarity. Branch tips are coloured according to year of isolation. To conserve space, isolate names have been abbreviated by removing the leading ‘20’ from each name, e.g. 2010–15611 is labelled as 10–15611. Distinctive metadata for each isolate are shown to the right of the tree, indicating geographical location of origin, *spa* type and presence of mobile elements, as indicated in the key above. All isolates carried the PVL phage φSa2wa-st5 [44] and SCC*mec*IVo [12] (Table S1, not shown in figure). (b) An unrooted version of tree in (a) to illustrate the clonal expansion of WA121 isolates with nodes coloured by year of isolation as in (a). (c) Unrooted tree constructed from high-quality SNPs identified between 2010–15611, 2012–18303 and diverse CC5/ST5 isolates as described in the text. TC-1024 (an abbreviation for TRIST CASE 1024) is a representative of a clonal outbreak of PVL-positive ST5-SCC*mec*IVo identified in the Australian Northern Territory and is part of the WA121 cluster described here. SNP differences between isolates and WA121 2010–15611 are shown in red text under each strain label.

­

During revision or production of Fig. 1b, the tree node label for strain WA121 isolate 2010–15611 (red circle with black outline) was separated from its correct branch tip. A corrected Fig. 1b with the 2010–15611 label in the correct position is included with this corrigendum. Additionally, during production, the isolate name 2010-15611 was incorrectly changed to "2010 to 15611" in the third paragraph of the results section.

